# Supplementary material for: The Effectiveness of Preoperative Outpatient and Home Rehabilitation and the Impact on the Results of Hip Arthroplasty: Introductory Report
Source: Healthcare (Basel). 2024 Jan 26;12(3):327. doi: 10.3390/healthcare12030327 (PMC10855594; doi:10.3390/healthcare12030327)
Supplement: Supplementary file 1 [file healthcare-12-00327-s001.zip › healthcare-2773832-supplementary.pdf]

Dear Patient,

thank You for participation in research. We hope the data received during the trial will make the treatment in the future more effective. You have been randomly selected to the group training at home. The proposed exercises are simple and can easily be applied at home.

Properly performed exercise should not exacerbate the pain or cause any other harm. Perform the exercises with convenient pace. Repeat every exercise 15-20 times. Try to relax and avoid excessive muscle tension. After the training You should feel a bit tired but not exhausted. You should not continue exercise if pain exacerbation occurred.

Please perform every exercise 5 times a week for next forthcoming 3 weeks. In case you are unable to perform the exercises for any reason please let me know on the next visit.

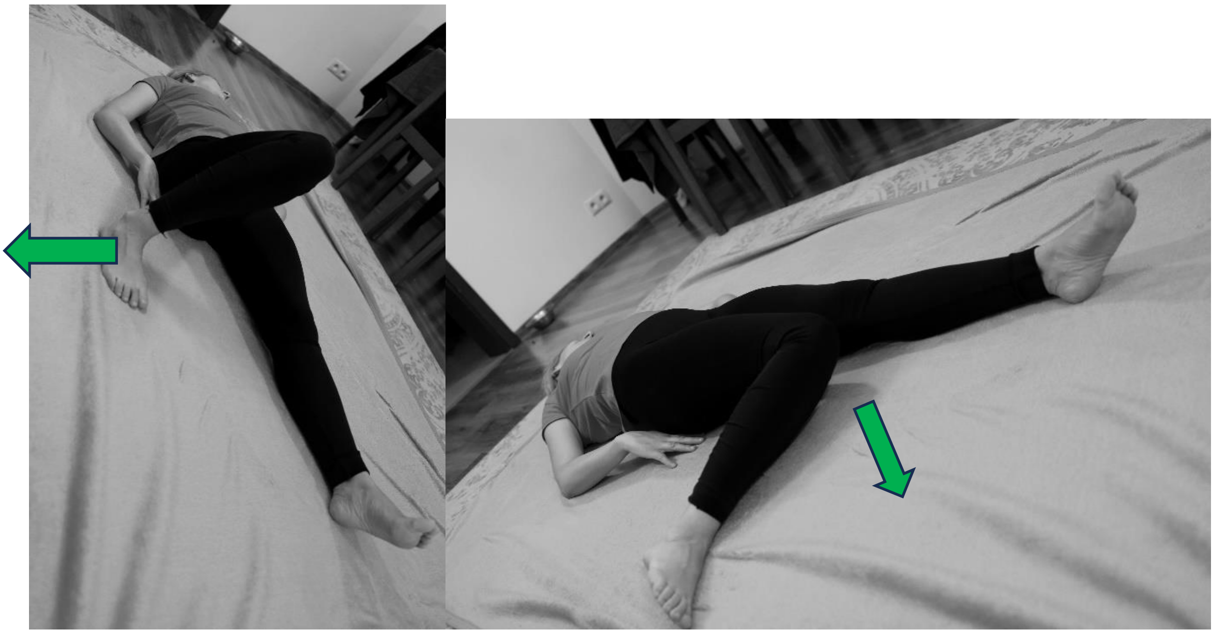

Lie on the back. Bend the hip and knee. Try to put your foot on the floor outside and try to move your knee close to the floor as much as you can. Do the same exercise with equal repetitions in unaffected leg.

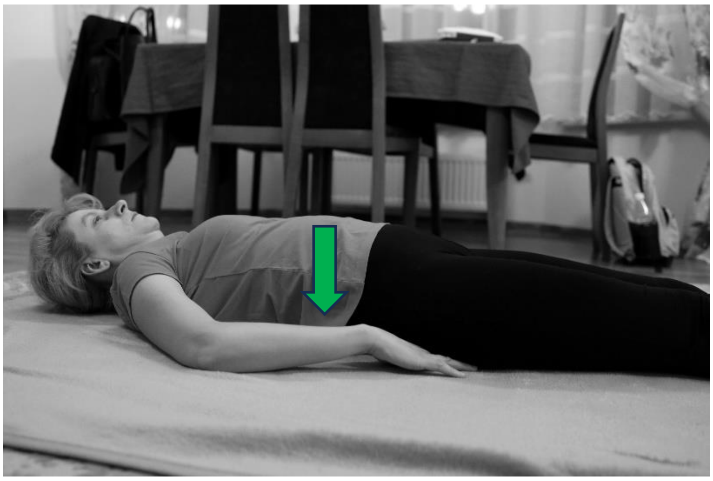

Tense your abdominal muscles the way to move your umbilicus down without moving your trunk. Keep the tension for 5-10 seconds.

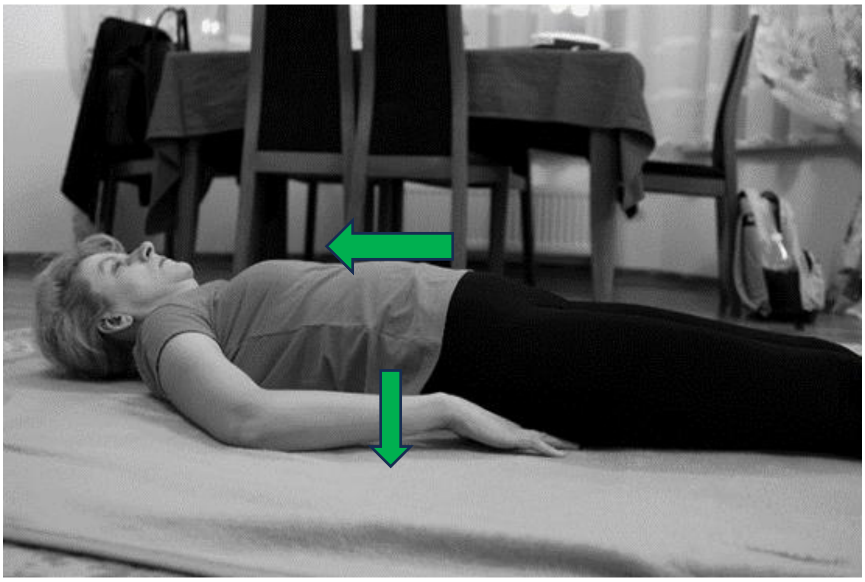

Use gluteal muscles to move the front of pelvis up. Try to touch the floor with your lumbar spine but avoid bending hips. Keep the tension for 5-10 seconds.

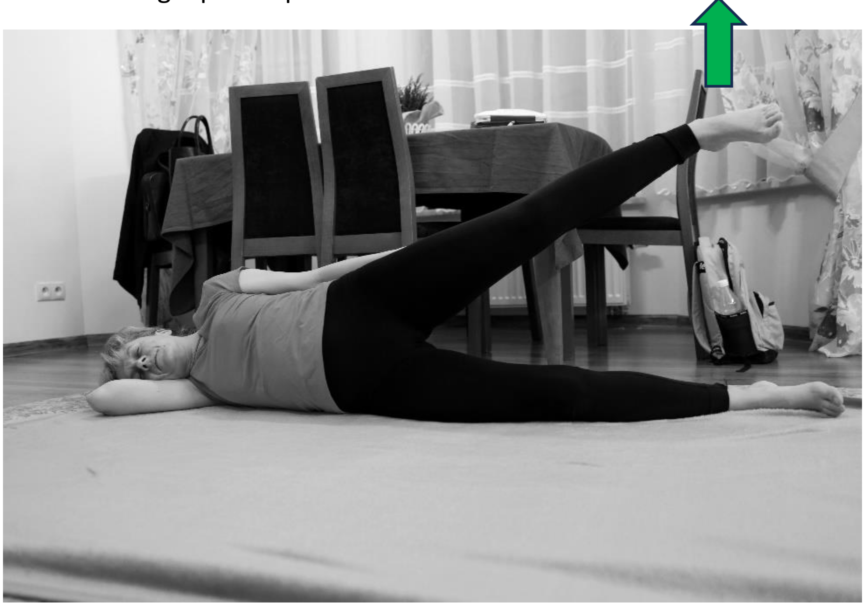

Lie on your side. Raise the leg as high as you can without engaging the trunk muscles. Keep the leg raised for 5-10 seconds. Do the same exercise with equal repetitions in unaffected leg.

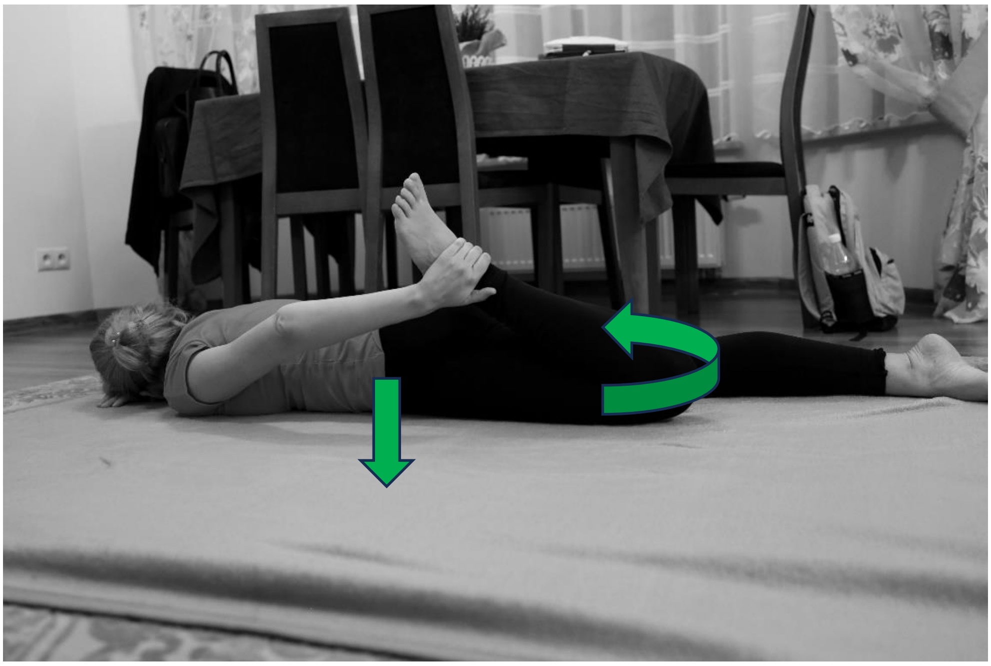

Lie on your front. Bend your knee as much as you can but without occurring pain. You can use hand to help you support the motion. Don’t let the pelvis raise from the floor. Try to remain in that position for 5-10 seconds. Do the same exercise with equal repetitions in unaffected leg.

In case of serious pain or need of any information do not hesitate to contact (my phone number is 603277722).
